# A Study on the Continuous and Discrete Wavelet Transform-Based Lithium-Ion Battery Fire Prediction Sensor Technology

**DOI:** 10.3390/s26051507

**Published:** 2026-02-27

**Authors:** Wen-Cheng Jin, Chang-Won Kang, Soon-Hyung Lee, Yong-Sung Choi

**Affiliations:** 1 Department of Electrical and Electronic Engineering, Dongshin University, Naju 58245, Republic of Korea; mskim0316@dsu.ac.kr (W.-C.J.); leehyung@dsu.ac.kr (S.-H.L.); 2 Department of Research Engineering PSD Enertech. Inc., Naju 58325, Republic of Korea; cwkang@psdtech.com

**Keywords:** lithium-ion battery, electromagnetic antenna sensor, high-frequency current transformer, wavelet transform, thermal runaway, micro internal short circuits

## Abstract

Early detection of fire-related risks in lithium-ion batteries (LIBs) remains a critical challenge, as conventional protection mechanisms typically activate only after irreversible degradation or macroscopic failure occurs. In this study, an innovative sensor-based diagnostic framework is proposed for proactive fire prediction in LIBs by simultaneously monitoring low-frequency and high-frequency electrical signatures generated during battery charge–discharge processes. An electromagnetic (EM) antenna sensor and a high-frequency current transformer (HFCT) sensor were employed to capture complementary voltage- and current-based transient signals associated with internal degradation phenomena. Cell-level experiments were conducted under various C-rates and temperature conditions, including high-stress environments, while module-level validation was performed on a 4-series, 1-parallel (4S1P) configuration at a 2C-rate under ambient temperature. Time–frequency characteristics of the measured signals were systematically evaluated using MATLAB-based continuous wavelet transform (CWT) and discrete wavelet transform (DWT) techniques. The results reveal that degradation-induced transient events exhibit non-stationary, impulsive voltage and current signatures with distinct frequency-band localization, which intensify with increasing C-rate, elevated temperature, and aging progression. At the module level, although signal amplitudes were partially attenuated due to current redistribution, characteristic wavelet energy patterns and time–frequency concentrations remained clearly distinguishable, demonstrating the scalability of the proposed approach. The combined EM antenna–HFCT sensing strategy, together with multi-resolution wavelet analysis, enables effective phenomenological differentiation between normal operational noise and incipient internal fault signatures well before conventional thermal or capacity-based indicators become evident. These findings demonstrate feasibility of the proposed method for early-stage fault diagnosis and highlight its potential applicability to advanced battery management systems for proactive fire prevention in large-scale energy storage and electric vehicle applications. Unlike conventional voltage-, temperature-, or gas-based diagnostics, the proposed approach enables the detection of incipient degradation phenomena at the microsecond scale by exploiting complementary low- and high-frequency electrical signatures. This study provides experimental evidence that wavelet-based EM and HFCT sensing can identify MISC-related precursors significantly earlier than conventional battery management indicators.

## 1. Introduction

Lithium-ion batteries (LIBs), owing to their high energy density, are the most commonly used secondary batteries and are widely employed not only in energy storage systems (ESSs) but also as power sources for electric vehicles (EVs). However, when accidents occur in systems utilizing LIBs, they can result in significant losses due to battery fires and explosions [1,2]. In the case of conventional fires, the use of appropriate firefighting equipment can suppress the fire before the combustible materials are completely consumed. In contrast, fires occurring in lithium-ion battery-based ESSs or EVs are driven by thermal runaway (TR) within the LIBs, in which internally generated heat undergoes self-amplification, leading to an uncontrollable sequence of heat generation, gas release, and subsequent fire or explosion. This mechanism progresses through a stepwise coupling of electrical, chemical, and thermal reactions [3,4,5,6]. In consideration of the fire hazards associated with LIBs, a wide range of studies on fire safety have been conducted. Representative examples include research on off-gas detection for LIBs [7] and experimental investigations on various fire suppression agents aimed at mitigating the propagation of TR in LIBs [8].

This paper introduces an innovative sensor-based fire prediction technology for LIBs that proactively mitigates fire risk by detecting electrical signatures generated during battery charge and discharge processes. The proposed approach integrates both low-frequency and high-frequency sensing modalities to capture a broad spectrum of degradation-related electrical phenomena under normal and defective operating conditions, thereby enabling early identification of fire-related hazards. Specifically, an electromagnetic (EM) antenna sensor is employed to monitor low-frequency EM emissions, while a high-frequency current transformer (HFCT) sensor is utilized to detect high-frequency transient current components. The degradation of LIBs is defined as the progressive deterioration of electrochemical performance and safety caused by repeated charge–discharge cycling, thermal exposure, voltage stress, and mechanical and chemical aging mechanisms. These degradation processes typically manifest as capacity fade, increased internal resistance, reduced power capability, and a heightened risk of TR. In this study, LIBs are conceptually modeled as an electrical insulation system, where the separator functions as the primary dielectric medium, enabling the interpretation of degradation-induced electrical anomalies as insulation-related phenomena. It should be emphasized that the term “PD-like” in this study refers to transient electrical signatures phenomenologically resembling partial discharge behavior, rather than gas-insulated or high-voltage insulation PD mechanisms. To effectively extract and characterize the non-stationary and transient electrical signatures associated with incipient failure, a MATLAB R2024a-based diagnostic framework employing continuous wavelet transform (CWT) and discrete wavelet transform (DWT) is developed, providing a robust time–frequency analysis tool for early fire risk prediction in LIB systems.

Accordingly, the objective of this study is to investigate whether degradation-induced micro internal short circuits (MISC) in LIBs can be reliably detected through non-intrusive electrical sensing and wavelet-based time–frequency analysis, prior to the onset of macroscopic failure or TR. To this end, this paper systematically analyzes low-frequency EM emissions and high-frequency current transients measured by an EM antenna and an HFCT sensor under various C-rates, temperatures, and aging conditions. It should be noted that this study is primarily exploratory and focuses on the phenomenological characterization of degradation-induced transient electrical signatures rather than statistical classification accuracy.

## 2. Methodology

### 2.1. Wavelet Transform

In general, signals can be classified into stationary and non-stationary signals. Among them, non-stationary signals are characterized by rapid variations in their frequency components, for which frequency-domain analysis based on the Fourier transform is not well suited. In contrast, WT methods are particularly effective for analyzing non-stationary signals, as they enable joint time–frequency analysis. WTs are broadly categorized into the CWT and the DWT, which are mathematically expressed in Equations (1) and (2), respectively.

(1) $$W^{f}\left(a,b\right)=<x\left(t\right),\psi_{a,b}\left(t\right)>\frac{1}{\sqrt{a}}\int_{-\infty }^{\infty }x\left(t\right)\psi^{*}\left(\frac{t-b}{a}\right)dt$$

(2) $$W^{f}\left(j,k\right)=<x\left(t\right),\psi_{j,k}\left(t\right)>\frac{1}{\sqrt{2^{j}}}\int_{-\infty }^{\infty }x\left(t\right)\psi^{*}\left(\frac{t-k2^{j}}{2^{j}}\right)dt$$

In Equation (1), a denotes the scale (dilation) parameter that determines the degree of compression or expansion, while b represents the translation parameter associated with shifts along the time axis. Using the mother wavelet (MW)  ψ(t), whose magnitude varies with scale, the CWT extracts scale-dependent components of the non-stationary signal x(*t*). However, the use of the CWT suffers from inherent redundancy and requires an infinite set of wavelets, leading to high computational complexity and substantial data requirements. In contrast, the DWT in Equation (2) suppresses redundancy through the use of orthogonal basis functions and enables efficient computation, particularly by employing a multi-resolution analysis (MRA) framework [9,10].

**Figure 1:**
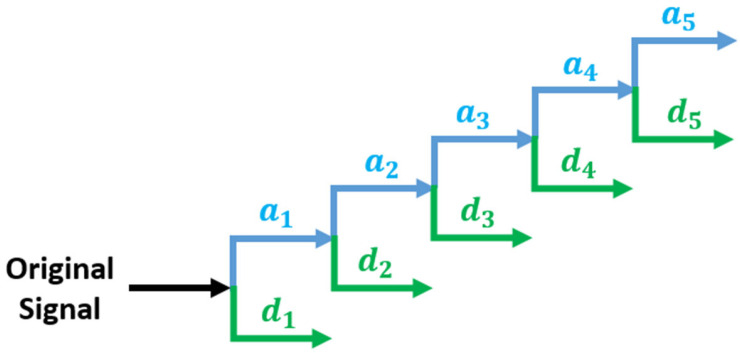
MRA for DWT (five level decomposition).

WT decomposes a signal through MRA into approximation coefficients obtained using a scaling function and detail coefficients derived from a MW. In this study, the decomposition level of the WT is treated as a variable parameter to determine the optimal WT configuration for detecting defect signals in LIBs. The multiresolution analysis procedure of the DWT is illustrated in Figure 1. As shown in Figure 1, the decomposition process is iteratively performed until the desired detail coefficients are obtained, where $a_{5}$ and $b_{5}$ represent the approximation and detail coefficients at level 5, respectively [11,12]. Furthermore, analytical methodologies previously applied to low-voltage direct current (LVDC) and micro-grid systems are extended and adapted to the battery domain in this work.

From the perspective of wavelet pattern analysis, the CWT yields continuous patterns in the time–frequency plane and is highly effective in identifying fine frequency shifts and the precise localization of transient events. Moreover, CWT results can be represented as images, known as scalograms, enabling intuitive pattern recognition. In other words, CWT provides insight into where and how a signal event occurs. In contrast, the DWT decomposes a signal into a set of discrete frequency bands across multiple levels, where patterns are characterized by variations in coefficient magnitude and energy, while temporal localization is provided in an approximate manner. That is, DWT primarily indicates in which frequency band a signal is present and how strong it is [13,14]. When comparing non-stationary signal analysis methods for LIB phenomena such as partial discharge (PD) and defect events, the CWT is primarily suited for pattern observation. Owing to its continuous scale variation, CWT provides precise temporal localization of event occurrences, facilitating structural understanding of the signal and physical interpretation. In particular, during PD events in LIBs, CWT enables accurate identification of the timing of high-frequency burst signals and is well suited for analyzing early-stage, subtle signals associated with MISC. In contrast, the DWT is mainly used for pattern quantification, as it decomposes signals into discrete frequency bands in a multilevel manner, yielding clear energy distributions across bands and making it highly effective for feature extraction and classification. Accordingly, PD levels can be classified and normal and defect operating conditions can be distinguished. While the CWT enables precise visualization of the time–frequency patterns of non-stationary signals through continuous scale analysis, the DWT efficiently quantifies the energy distribution of signals via discrete frequency decomposition, making it well suited for automated diagnostic algorithms [15,16,17,18]. Table 1 summarizes the differences in pattern analysis between CWT and DWT.

Representative MWs used in the DWT include Daubechies (dbN), Symlet (symN), Coiflet (coifN), and biorthogonal (bior) wavelets, whereas commonly employed MWs for the CWT include Morlet (amor), Mexican hat (mexh), Paul wavelet, and Gaussian (gausN) wavelets. These MWs are classified according to their characteristics and research objectives, as summarized in Table 2.

DWTs employ filter-bank-based MWs optimized for feature extraction, whereas CWTs utilize physically interpretable MWs, such as the Morlet wavelet, to provide high-resolution time–frequency representations of non-stationary transient signals. Furthermore, the Morlet-based CWT effectively reveals microsecond-scale transient signatures that are not observable in conventional fast fourier transform (FFT) spectra, enabling early detection of MISC-related electrical anomalies in LIBs.

In this study, the CWT is primarily employed to visualize the temporal occurrence and frequency localization of degradation-induced transient events, whereas the DWT is utilized to quantify energy distribution across frequency bands for comparative analysis under different operating conditions. The Morlet wavelet was selected for CWT due to its Gaussian-modulated sinusoidal structure, which closely resembles EM transient waveforms. The Daubechies (dbN) wavelet was selected for DWT as it provides a suitable balance between temporal localization and robustness to high-frequency noise, while maintaining computational efficiency for potential real-time implementation.

### 2.2. Assimilation of Long- and Short-Wavelength Information

In this study, long-wavelength (low-frequency) and short-wavelength (high-frequency) components are not combined through an explicit weighted summation model, but rather assimilated through a hierarchical diagnostic framework. The DWT-based energy ratios at selected decomposition levels (e.g., D1/D_total and D4–D8/D_total) are first computed independently for high- and low-frequency bands.

Instead of applying arbitrary weighting coefficients, the diagnostic interpretation is based on relative dominance patterns across scales. Specifically, high D1–D2 energy concentration is interpreted as high-frequency transient dominance (associated with localized MISC-like events), whereas elevated D4–D8 energy indicates low-to-mid-frequency EM behavior linked to global system dynamics.

Preliminary offline experiments were conducted under controlled C-rate and temperature variations to determine stable threshold ranges of relative energy concentration. These experiments were designed to compare normal operation, moderate degradation, and high-stress conditions, thereby establishing reference patterns rather than absolute weighted fusion parameters.

Therefore, the assimilation strategy is rule-based and scale-discriminative rather than weight-optimized, which enhances interpretability and reduces overfitting risk for real-time BMS implementation.

### 2.3. Lithium-Ion Battery Degradation and Thermal Runaway

TR in LIBs remains one of the most critical safety challenges in EVs and ESSs. While traditional studies focus on abuse-induced short circuits, recent experimental evidence suggests that MISC acts as an early-stage trigger during long-term degradation. MISC is increasingly recognized as a critical precursor to TR in LIBs, particularly under aging and abusive conditions. Unlike macroscopic internal short circuit (ISC), MISC is characterized by localized, low-current, and intermittent electrical pathways formed due to separator degradation, lithium dendrite penetration, or particle detachment [19,20].

The definition and characteristics of MISC are as follows [21,22]:

#### 2.3.1. Definition

MISC refers to microscale, localized, and often intermittent ISCs occurring within a lithium-ion cell, typically involving:

- Partial separator thinning or pinhole formation;
- Lithium dendrite bridging;
- Conductive debris from electrode fracture.

#### 2.3.2. Key Characteristics

The origin of MISC in battery degradation are as follows:

#### 2.3.3. Separator Degradation [23,24]

Separator degradation is a primary origin of MISC in LIBs. Thermal shrinkage of the polymer separator under elevated temperature conditions reduces pore integrity and thickness, increasing the probability of direct electronic contact between electrodes. In addition, chemical oxidation induced by reactive species released from cathode decomposition progressively weakens the separator matrix. Mechanical stresses generated during repeated charge–discharge cycling further lead to the formation of microcracks and pinholes, which act as preferential pathways for localized short-circuit initiation.

#### 2.3.4. Lithium Plating and Dendrite Growth [25,26]

Lithium plating and subsequent dendritic growth are strongly associated with operating conditions such as low-temperature charging and high-rate charging, where lithium intercalation kinetics are limited. Non-uniform current distribution across the electrode surface promotes localized lithium deposition, while repeated rupture and regrowth of the solid electrolyte interphase (SEI) create electrically conductive lithium filaments. These dendrites can penetrate the separator and form transient or persistent micro-scale internal short-circuit paths.

#### 2.3.5. Electrode Particle Fracture [27,28,29]

Electrode particle fracture arises from volume-change-induced mechanical stress during lithium insertion and extraction. Cracked or fragmented active material particles may become electrically isolated from the electrode matrix and migrate toward the separator. The intrusion of conductive particle debris into separator pores significantly increases the likelihood of microscale electronic bridging between the anode and cathode, thereby contributing to the formation of MISC.

The electro-thermal coupling mechanism of MISC is as follows:

#### 2.3.6. Localized Joule Heating [30,31]

The presence of a MISC leads to localized heat generation governed by Joule heating, which can be expressed as Equation (3).

(3) $$Q_{MISC}=I_{MISC}^{2}R_{local}$$

Although the current associated with MISC is relatively small compared to that of a hard ISC, the heat is generated within an extremely confined region. As a result, even low-level MISC currents can produce a substantial local temperature rise, since the generated heat is concentrated in a microscale volume rather than being distributed throughout the cell.

#### 2.3.7. Thermal Accumulation [6,32,33]

The separator and electrolyte exhibit intrinsically low thermal conductivity, which significantly limits heat dissipation from the MISC region. When MISC events occur repeatedly or intermittently, the generated heat cannot be fully released between events, leading to progressive thermal accumulation. Consequently, the local temperature in the vicinity of the MISC can exceed the average bulk cell temperature by approximately 10–50 °C, creating favorable conditions for accelerated degradation reactions and eventual TR initiation.

The transition from MISC to TR is shown in Table 3:

Table 4 highlights the sequential thermal degradation stages of a lithium-ion battery. The process begins with the breakdown of the Solid Electrolyte Interphase (SEI) layer at relatively low temperatures (90–120 °C), followed by the melting of the separator, which can lead to macroscopic internal short circuits. As the temperature rises further, electrolyte oxidation occurs, eventually leading to oxygen release from the cathode material, which triggers uncontrollable thermal runaway.

In general, the degradation process from MISC of LIBs can be approached in three ways from an electrical signal perspective.

#### 2.3.8. Electrical Signatures [34]

MISCs manifest electrically as transient and irregular signal disturbances rather than steady-state abnormalities. Typical electrical signatures include high-frequency voltage spikes, microsecond-scale current fluctuations, and increased common mode voltage (CMV) noise. These signals arise from the intermittent and localized nature of MISC events, which cause rapid changes in current paths and local impedance within the cell.

#### 2.3.9. Thermal Signatures [35]

From a thermal perspective, MISC induces abnormal temperature behavior that deviates from normal operating trends. Localized Joule heating leads to an increased temperature rise rate (*dT*/*dt*), which cannot be explained by global cell loading conditions. In addition, infrared thermography often reveals localized hot spots near the MISC region, indicating highly non-uniform internal heat generation.

#### 2.3.10. Diagnostic Implications [36]

Due to the transient and non-stationary characteristics of MISC-related signals, conventional time-domain or frequency-domain analysis methods are often insufficient for reliable detection. Wavelet-based time–frequency analysis provides an effective diagnostic approach by isolating MISC-induced transient signatures across multiple scales, thereby enabling early discrimination of incipient ISCs from background noise and normal operational disturbances.

MISC represents a hidden but critical pathway linking battery degradation to TR. Through localized Joule heating, electro-thermal feedback, and chemical reaction acceleration, MISC gradually drives the cell toward catastrophic failure. Early detection and mitigation of MISC are therefore essential for next-generation battery safety management systems.

Therefore, the electrical characteristics of MISC provide a strong physical basis for the application of wavelet-based analysis to sensor signals measured during battery operation, which motivates the experimental and analytical framework adopted in this work.

## 3. Experimental

In this paper, cylindrical 18650 LIBs manufactured by Samsung SDI (Samsung SDI; Yongin-si, Gyeonggi-do, Republic of Korea), with a rated capacity of 3000 mAh and a nominal voltage of 3.6 V, were used. Table 5 summarizes the specifications of the batteries used in this work. Battery degradation experiments were conducted under both room-temperature (25 °C) and high-temperature (60 °C) conditions.

Cell-level tests were performed at 1C, 2C, and 5C-rates, where the cells were charged to 4.2 V using a constant current–constant voltage (CCCV) protocol and subsequently discharged to 2.5 V under constant current (CC) mode. In addition, a simulation study was carried out to extend the analysis to a module-level configuration consisting of a 4-series, 1-parallel (4S1P) module operated at a 2C-rate under room-temperature conditions. A total of 60 cells were tested, corresponding to 10 cells per operating condition, and each experiment was repeated at least three times to verify repeatability. For module-level validation, 5 modules were evaluated under identical protocols.

During each degradation test, a NEWARE CT-4008 (NEWARE; Shenzhen, China) battery tester was used for charge–discharge control, and frequency-domain measurements were acquired using a PicoScope 2000 (Pico Technology; Cambridgeshire, UK) series oscilloscope. During the degradation test, the sampling rate to 0.050 μs, and the CMV trigger thresholds were configured as Max 180 mV to Min −150 mV. The sensors used were EM wave antenna (1–30 MHz) and HFCT (1–500 MHz) sensors. During the degradation test, the sampling rate was set to 0.050 μs (corresponding to a 20 MHz sampling rate), corresponding to a maximum detectable frequency of 10 MHz, ensuring sufficient resolution for capturing fast transient events.

**Figure 2:**
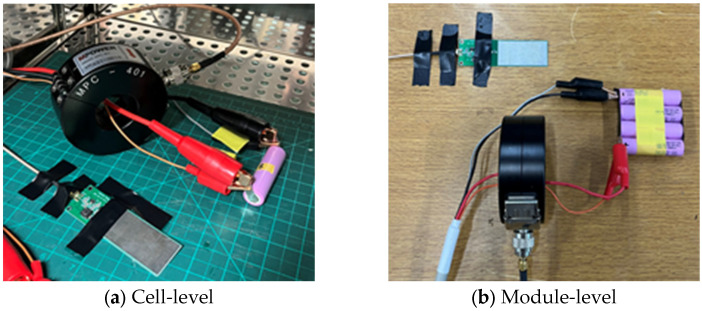
Test sensor configuration.

In this study, signals detected during the degradation process of LIBs under various operating conditions were analyzed using MATLAB-based CWT and DWT pattern analysis. The Morlet wavelet was used as the MW for the CWT, while Daubechies wavelets (dbN) were used for the DWT. By systematically varying the decomposition level and order of the MWs, the optimal wavelet configuration for detecting MISC signals in LIBs was identified. Figure 2 below shows the sensor configuration. For the DWT analysis, decomposition levels from 1 to 8 were evaluated, and db4 was selected as a representative compromise between temporal resolution and noise robustness. Preliminary comparison with db2, db6, and sym4 indicated that db4 provided superior transient isolation while minimizing coefficient spreading across adjacent levels. In particular, db2 exhibited excessive sensitivity to high-frequency noise, whereas db6 and sym4 produced broader coefficient distributions that reduced temporal localization sharpness. Therefore, db4 was selected as the optimal MW for balancing transient resolution, noise suppression, and computational efficiency. The superiority of db4 was assessed based on coefficient sparsity, transient peak localization sharpness, and minimal energy leakage to adjacent decomposition levels.

## 4. Results and Discussion

### 4.1. Cell-Level Results

In this study, the degradation behavior of LIBs was investigated under room-temperature (25 °C) and high-temperature (60 °C) conditions. As cycling progressed, the battery capacity gradually decreased, while the rate of capacity fade became significantly accelerated under high-temperature and high C-rate conditions due to the enhancement of parasitic side reactions and the decomposition of the SEI.

**Figure 3:**
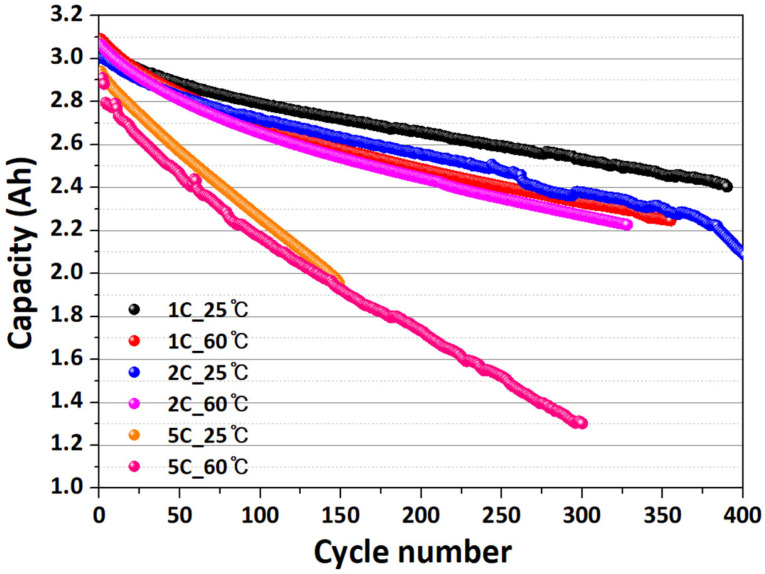
Capacity change according to degradation experiment.

Figure 3 illustrates the evolution of battery capacity during the degradation tests. As shown in Figure 3, the most pronounced capacity degradation was observed under the 5 C-rate test at elevated temperature, confirming that thermal stress combined with high current density markedly accelerates LIB degradation.

**Figure 4:**
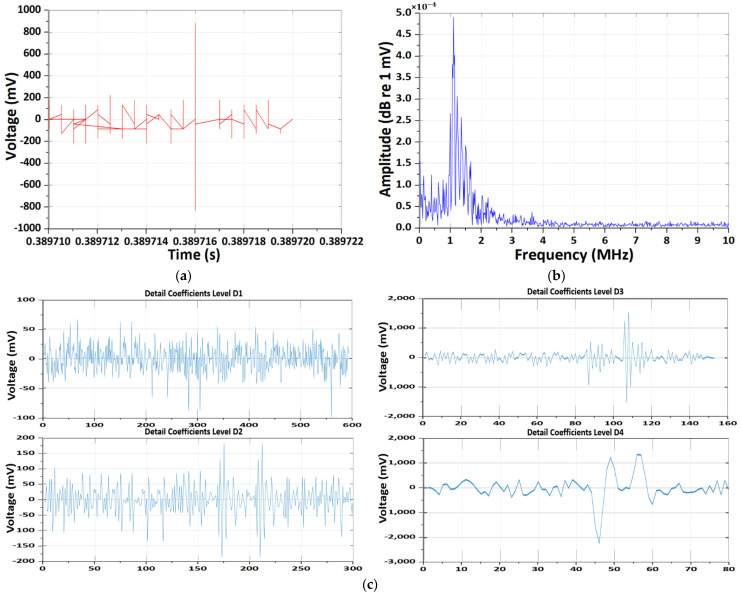

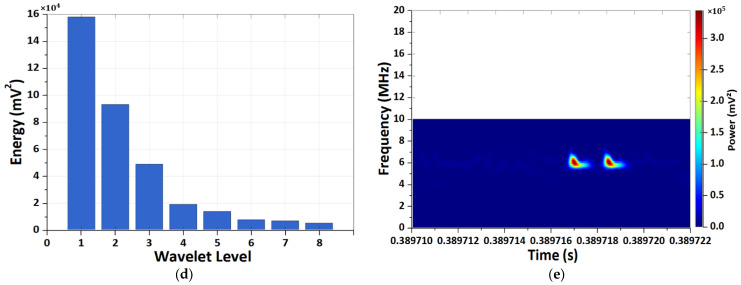
Results of the CWT and DWT pattern analyses of EM antenna sensor measurement CMV noise. (**a**) Results of time-domain CMV signal. (**b**) Results of frequency-domain spectrum. (**c**) Results of DWT detail coefficients at decomposition levels D1–D4. (**d**) Results of energy distribution across decomposition levels of the Daubechies wavelet. (**e**) Results of Morlet continuous wavelet time–frequency spectrum.

The results of the CWT and DWT pattern analyses of EM antenna sensor measurement CMV noise throughout the overall testing process are presented in Figure 4. During the initiation of CCCV charging using the NEWARE CT-4008 system, a pronounced transient EM response was detected by the EM antenna. In the time-domain signal, a large impulse with sub-microsecond rise time was observed, corresponding to the activation of the constant-current control loop. The FFT spectrum shows dominant energy below 2 MHz, which reflects the time-averaged response of the system-level transient rather than repetitive discharge phenomena.

The wavelet-based analysis further reveals that the transient energy is mainly concentrated in intermediate frequency scales. Notably, the Morlet CWT time–frequency spectrum exhibits two distinct high-energy bursts in the 5–6 MHz range. These two events are attributed to successive control actions during the CCCV charging initiation, including the rapid current ramp-up and subsequent voltage loop stabilization, as well as the re-excitation of parasitic LC resonance formed by the charger, cables, and battery input impedance.

Therefore, the observed dual energy bursts are not indicative of PD activity, but rather represent EM interference generated by the power-electronic charging system during transient operating conditions.

**Figure 5:**
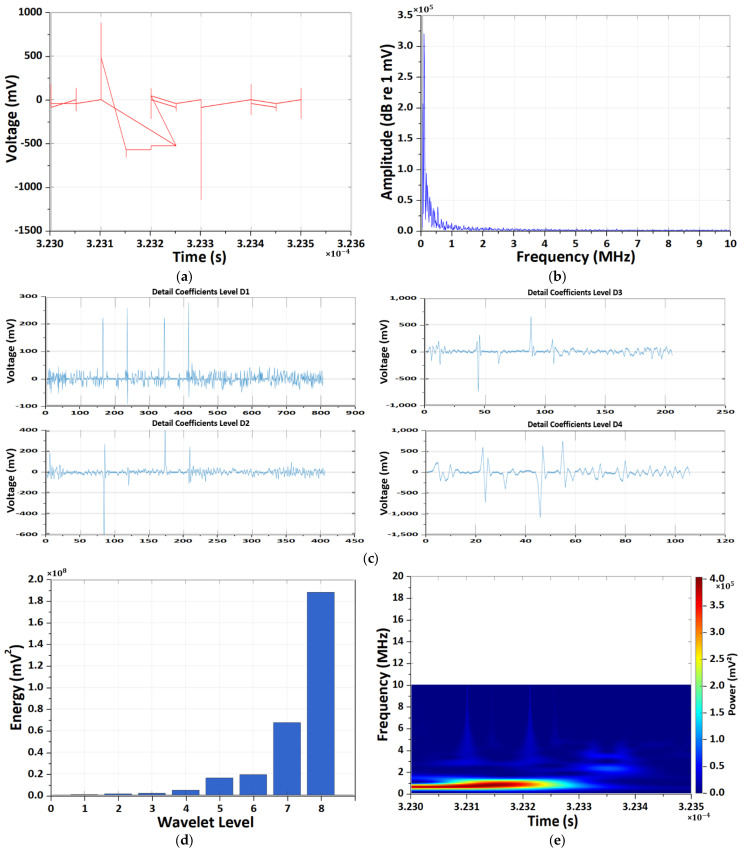
Results of the CWT and DWT pattern analyses of EM antenna sensor measurement under the 1 C-rate high-temperature condition. (**a**) Results of time-domain EM PD signal. (**b**) Results of frequency-domain spectrum. (**c**) Results of DWT detail coefficients at decomposition levels D1–D4. (**d**) Results of energy distribution across decomposition levels of the Daubechies wavelet. (**e**) Results of Morlet continuous wavelet time–frequency spectrum.

**Figure 6:**
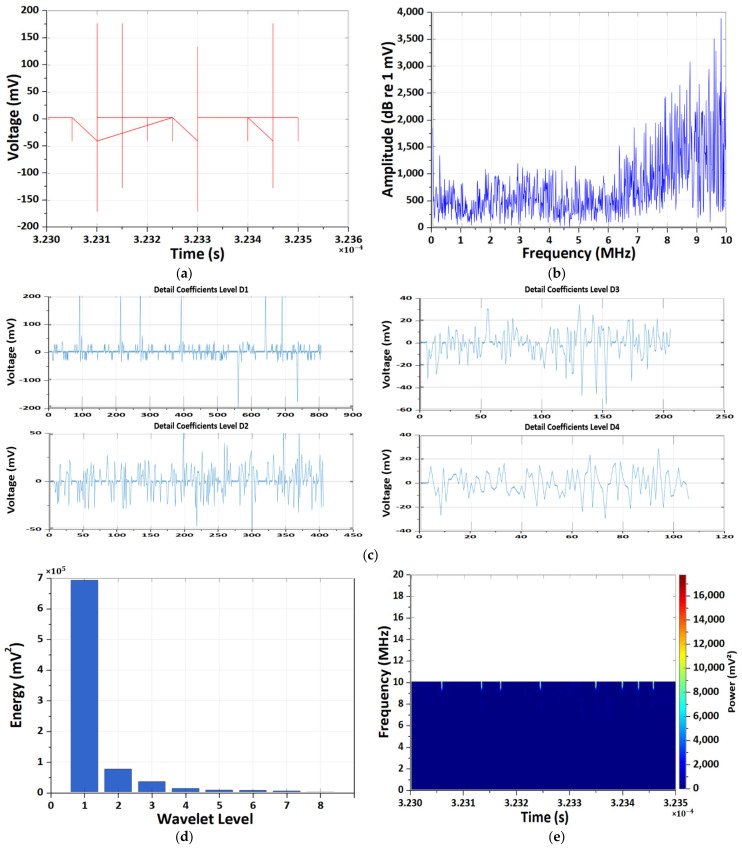
Results of the CWT and DWT pattern analyses of HFCT sensor measurement under the 1 C-rate high-temperature condition. (**a**) Results of time-domain PD signal. (**b**) Results of frequency-domain spectrum. (**c**) Results of DWT detail coefficients at decomposition levels D1–D4. (**d**) Results of energy distribution across decomposition levels of the Daubechies wavelet. (**e**) Results of Morlet continuous wavelet time–frequency spectrum.

Figure 5 and Figure 6 present a comparative analysis of EM antenna and HFCT measurements obtained under the 1C-rate high-temperature (60 °C) condition. Although both sensing modalities detect degradation-induced transient behavior, their dominant time–frequency characteristics exhibit clear distinctions.

The EM antenna measurements (Figure 5) reveal repeatedly occurring abnormal voltage spikes in the time domain, with amplitudes reaching several hundred millivolts and sub-microsecond durations. These non-periodic and asymmetric waveforms indicate stochastic PD-like disturbances rather than switching-related noise. The corresponding FFT spectrum shows dominant energy concentration in the low-to-mid frequency range (several hundred kilohertz to a few megahertz), with gradual attenuation toward higher frequencies. DWT analysis further indicates that the primary energy is concentrated at intermediate decomposition levels (notably D4–D8), reflecting low-frequency-dominant transient activity. The Morlet CWT scalogram confirms strong time–frequency localization around approximately 1 MHz during discharge occurrence intervals, suggesting low-to-mid-frequency electromagnetic emissions associated with degradation processes.

In contrast, HFCT measurements (Figure 6) exhibit sharply localized bipolar impulsive waveforms with peak amplitudes of approximately ±150–180 mV and sub-microsecond widths, indicative of rapid internal current transients. The frequency-domain spectrum extends up to the upper measurement bandwidth (~10 MHz), with pronounced spectral energy above 6–7 MHz. Consistently, DWT energy is overwhelmingly concentrated at the first decomposition level (D1), demonstrating strong high-frequency dominance. The Morlet CWT scalogram reveals intermittent, high-power bursts localized around 9–10 MHz, clearly separated in time and exhibiting discharge-like characteristics.

Overall, the comparative analysis under 1C high-temperature conditions demonstrates that EM antenna sensing primarily captures low-to-mid-frequency voltage-based PD-like signatures, whereas HFCT sensing selectively resolves high-frequency, current-based impulsive transients associated with localized degradation phenomena. The complementary time–frequency characteristics observed in DWT and CWT analyses confirm that multi-sensor wavelet-based diagnostics provide enhanced discrimination capability between system-level EM behavior and internal MISC-related transient events.

**Figure 7:**
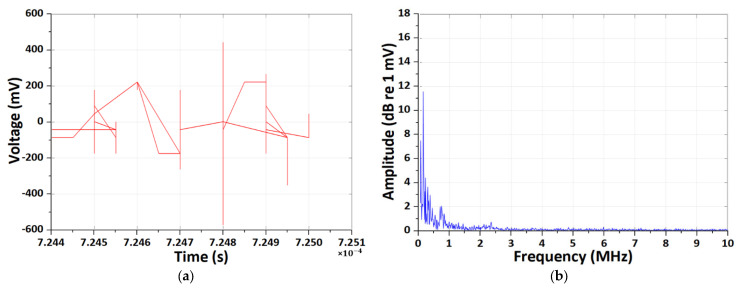

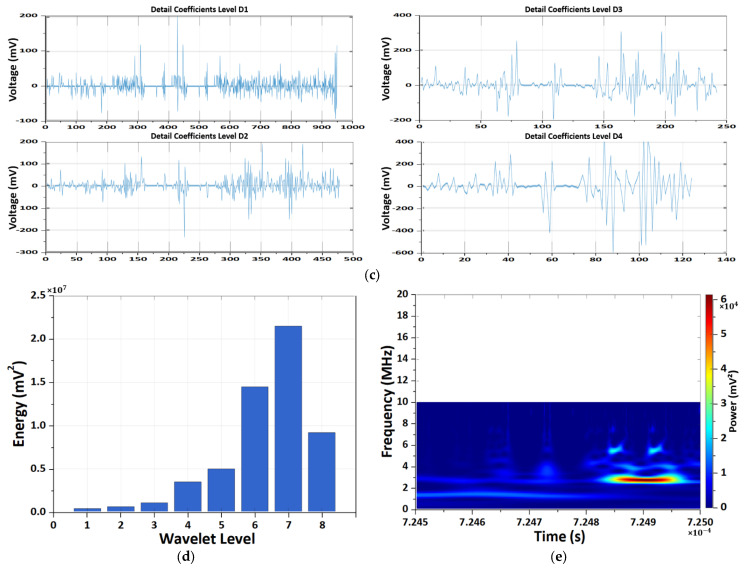
Results of the CWT and DWT pattern analyses of EM antenna sensor measurement under the 2 C-rate room-temperature condition. (**a**) Results of time-domain EM PD signal. (**b**) Results of frequency-domain spectrum. (**c**) Results of DWT detail coefficients at decomposition levels D1–D4. (**d**) Results of energy distribution across decomposition levels of the Daubechies wavelet. (**e**) Results of Morlet continuous wavelet time–frequency spectrum.

Similar results were obtained under other test conditions as well. Figure 7 illustrates the EM antenna-based signal analysis of a LIB during the degradation process under a 2C condition at 25 °C, including time-domain, frequency-domain, DWT and CWT representations.

The time-domain waveform exhibits comparatively slower-varying and larger-amplitude fluctuations, with intermittent impulsive excursions reaching several hundred millivolts, superimposed on a non-zero baseline, reflecting a mixture of transient disturbances and load-dependent EM emissions. In contrast to HFCT measurements, these signals show less sharply localized impulses and more pronounced low-frequency components, indicating strong coupling between the antenna sensor and the global current and voltage dynamics of the battery system.

The corresponding frequency-domain spectrum is dominated by low-frequency content below approximately 1 MHz, where spectral amplitudes are significantly higher and gradually decay with increasing frequency, suggesting that the measured emissions are primarily associated with switching harmonics, current ripple, and slow transient phenomena rather than purely high-frequency discharge events.

DWT analysis reveals that the signal energy is concentrated at higher decomposition levels, particularly levels D6–D8, confirming that the dominant energy resides in lower-frequency bands with broader temporal support.

Time-domain inspection of the DWT detail coefficients further demonstrates that higher-level coefficients exhibit large-amplitude, slowly varying oscillations, while lower-level coefficients contain relatively weaker and sparsely distributed high-frequency components.

Finally, the Morlet CWT scalogram shows sustained time–frequency energy concentrations mainly in the low- to mid-frequency range (approximately 2–5 MHz), with extended temporal duration rather than isolated bursts, indicating quasi-continuous EM emissions during the charge–discharge process.

Overall, these results demonstrate that EM antenna measurements under moderate C-rate conditions predominantly capture low-frequency, system-level EM signatures associated with battery operation and degradation, highlighting a clear contrast with the impulsive, high-frequency characteristics observed in HFCT-based partial-discharge-oriented measurements.

**Figure 8:**
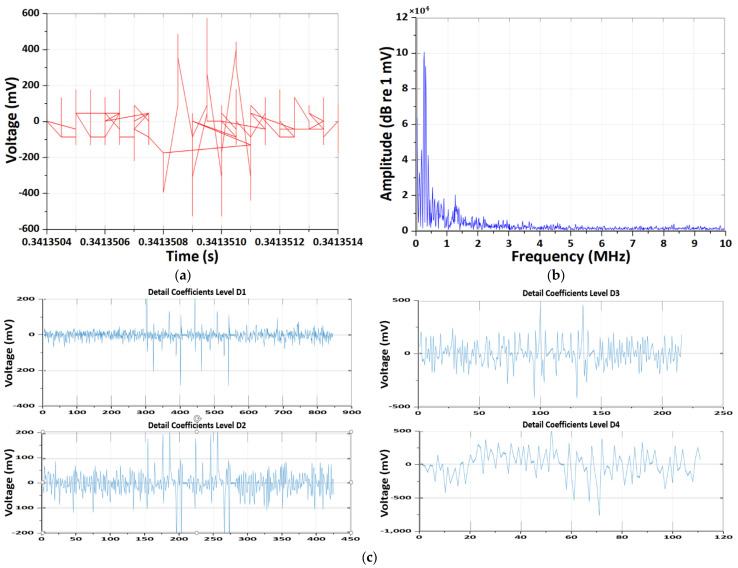

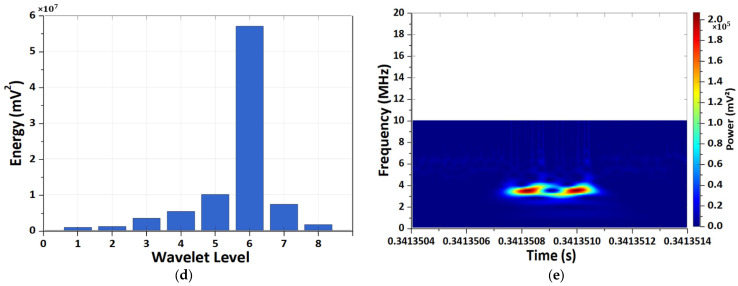
Results of the CWT and DWT pattern analyses of EM antenna sensor measurement under the 5 C-rate high-temperature condition. (**a**) Results of time-domain EM PD signal. (**b**) Results of frequency-domain spectrum. (**c**) Results of DWT detail coefficients at decomposition levels D1–D4. (**d**) Results of energy distribution across decomposition levels of the Daubechies wavelet. (**e**) Results of Morlet continuous wavelet time–frequency spectrum.

**Figure 9:**
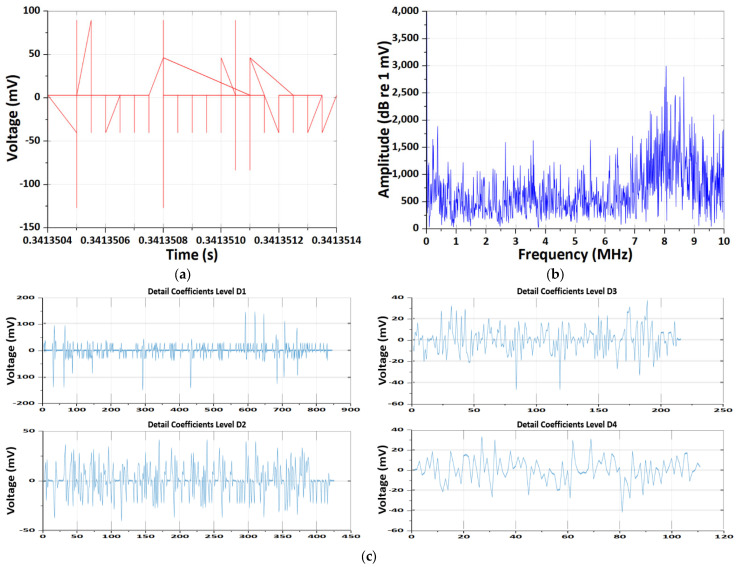

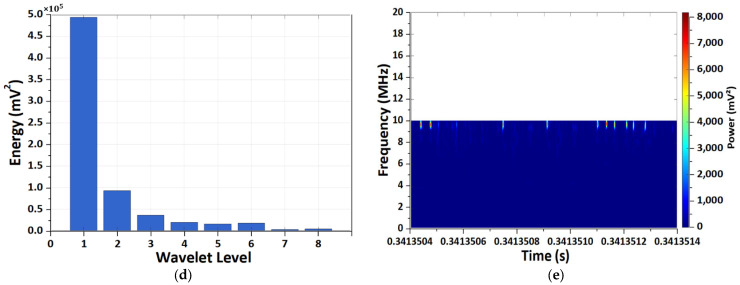
Results of the CWT and DWT pattern analyses of HFCT sensor measurement under the 5 C-rate high-temperature condition. (**a**) Results of time-domain PD signal. (**b**) Results of frequency-domain spectrum. (**c**) Results of DWT detail coefficients at decomposition levels D1–D4. (**d**) Results of energy distribution across decomposition levels of the Daubechies wavelet. (**e**) Results of Morlet continuous wavelet time–frequency spectrum.

Notably, the same trends were consistently observed under the worst-case condition of a 5 C-rate at high temperature, indicating the robustness and reproducibility of the experimental results. Figure 8 and Figure 9 present a comprehensive comparison of EM antenna and HFCT-based measurements acquired from a LIB operating under the most severe degradation condition, namely a 5C-rate at high temperature.

The EM antenna measurements (Figure 8) displayed large-amplitude, non-stationary voltage fluctuations accompanied by baseline drift and temporally extended transients. The corresponding frequency spectra exhibited broadband energy distributions primarily concentrated in the low-to-mid frequency region. DWT analysis revealed distributed energy across multiple decomposition levels (D4–D7), indicating the coexistence of operational noise and mid-frequency electromagnetic radiation. The Morlet CWT scalogram further confirmed temporally extended energy bands, reflecting strong sensitivity to system-level electromagnetic emissions induced by aggressive current flow and converter switching.

In contrast, HFCT measurements (Figure 9) were characterized by sharply localized, bipolar impulsive waveforms with steep rise times, indicative of rapid internal current transients. The frequency-domain spectra emphasized high-frequency components near the upper measurement bandwidth. Consistently, DWT energy was strongly concentrated in the lowest decomposition levels (D1–D2), confirming high-frequency dominance. The Morlet CWT scalogram revealed discrete, high-power bursts localized near the upper frequency range, consistent with discharge-like transient events associated with internal degradation mechanisms.

Overall, these results demonstrate that under extreme 5C-rate high-temperature conditions, EM antenna sensing predominantly captures global EM behavior associated with severe operating stress, while HFCT sensing selectively resolves localized, high-frequency transient phenomena linked to internal degradation mechanisms, underscoring the complementary diagnostic capabilities of the two sensors for advanced battery health monitoring.

These results indicate that degradation-induced transient signatures become increasingly prominent under elevated temperature and high C-rate conditions, supporting the hypothesis that electrical sensing can serve as an early indicator of internal fault evolution.

### 4.2. Module-Level Results

Based on the cell-level test results, CWT and DWT pattern analyses were further conducted at the module level using a four-series, one-parallel (4S1P) configuration. This extension demonstrates that the proposed signal analysis framework is not limited to cell-level evaluation but can be effectively scaled to realistic EV and ESS applications, thereby establishing a foundation for practical, real-world implementation.

**Figure 10:**
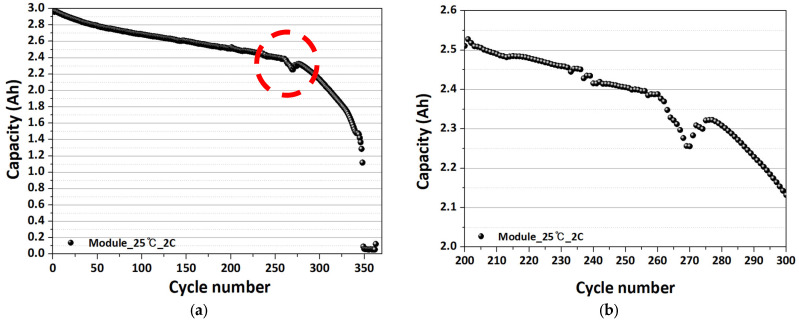
Capacity change according to degradation experiment at module level. (**a**) Charge–discharge behavior over the full cycling range. (**b**) Enlarged view of a specific interval highlighted in (**a**).

Figure 10 below shows the degradation behavior of a 4S1P LIB module evaluated under a 2C-rate condition at room temperature. The capacity retention profile exhibits an initially gradual and near-linear decline over the early cycling stage, indicating that conventional aging mechanisms, such as SEI growth and progressive loss of active lithium, dominate the degradation process. As cycling proceeds beyond approximately 250 cycles, the capacity fade rate increases noticeably, marking the onset of an accelerated degradation regime at the module level. In this region, intermittent capacity fluctuations and partial recovery behavior are observed, which can be attributed to evolving cell-to-cell imbalance, increased polarization, and transient redistribution of lithium among series-connected cells. At later stages, the capacity decreases monotonically without recovery, suggesting irreversible structural degradation, internal resistance growth, and loss of electrochemically active material.

Overall, these results demonstrate that even under moderate operating conditions, a 4S1P module exhibits non-linear degradation characteristics that emerge earlier than typically observed in single-cell tests, underscoring the importance of module-level diagnostics for early detection of abnormal aging and reliability degradation.

**Figure 11:**
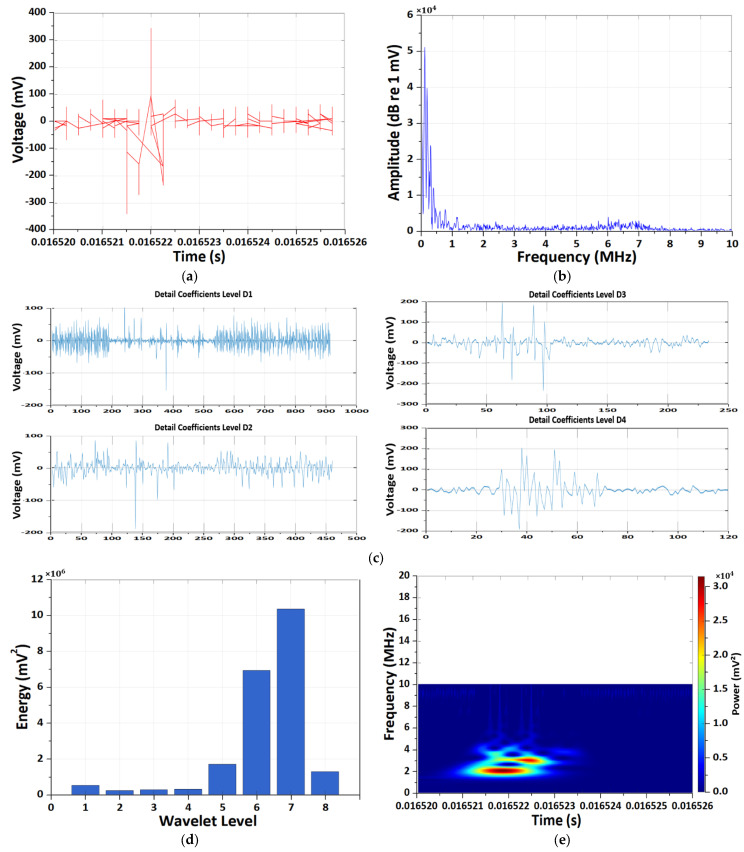
Results of the CWT and DWT pattern analyses of EM antenna sensor measurement 4S1P LIB module operated at a 2C-rate under room-temperature condition. (**a**) Results of time-domain EM PD signal. (**b**) Results of frequency-domain spectrum. (**c**) Results of DWT detail coefficients at decomposition levels D1–D4. (**d**) Results of energy distribution across decomposition levels of the Daubechies wavelet. (**e**) Results of Morlet continuous wavelet time–frequency spectrum.

**Figure 12:**
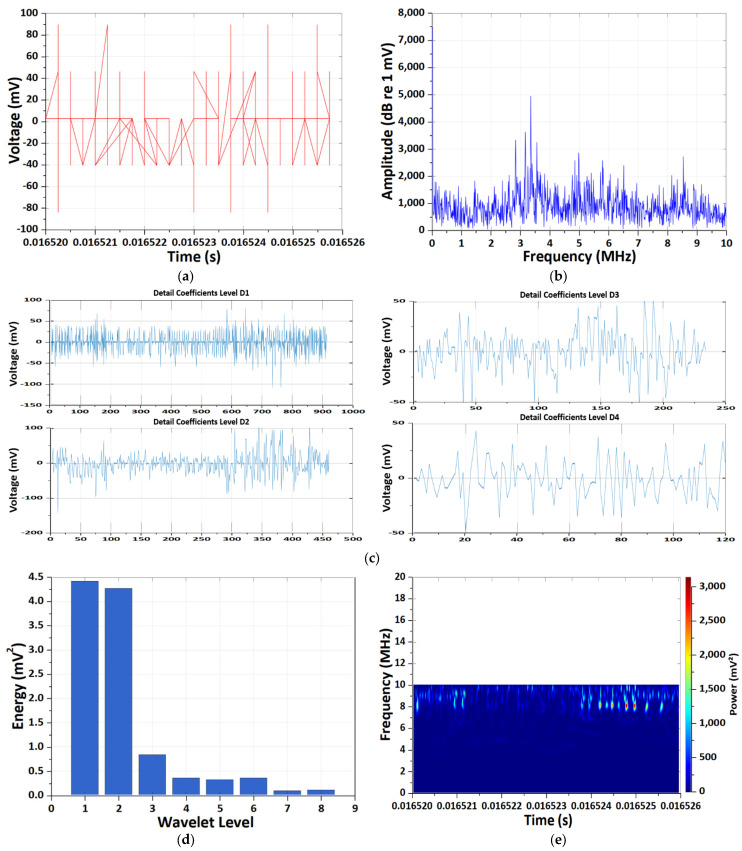
Results of the CWT and DWT pattern analyses of HFCT sensor measurement 4S1P LIB module operated at a 2C-rate under room-temperature condition. (**a**) Results of time-domain PD signal. (**b**) Results of frequency-domain spectrum. (**c**) Results of DWT detail coefficients at decomposition levels D1–D4. (**d**) Results of energy distribution across decomposition levels of the Daubechies wavelet. (**e**) Results of Morlet continuous wavelet time–frequency spectrum.

Figure 11 and Figure 12 present a comparative analysis of EM antenna and HFCT-based measurements obtained from a 4S1P LIB module operated at a 2C-rate under room-temperature conditions.

In Figure 11, the EM antenna measurements exhibit relatively smooth yet non-stationary voltage fluctuations with intermittent impulsive excursions, reflecting strong sensitivity to system-level EM emissions arising from module-scale current redistribution, interconnection impedance, and switching-related noise.

The corresponding frequency spectra are dominated by low-to-mid-frequency components, and the DWT energy is distributed over higher decomposition levels, indicating that the measured signals are primarily governed by slower, spatially extended EM phenomena rather than sharply localized transients.

By contrast, the HFCT measurements in Figure 12 reveal more distinct impulsive features with clearer temporal localization, although their amplitudes and occurrence rates are notably reduced compared to cell-level observations.

The frequency-domain characteristics emphasize higher-frequency components, and the DWT energy is concentrated in the lower decomposition levels, confirming the presence of localized current transients within the module.

The Morlet CWT scalograms further highlight this distinction: while EM antenna signals display temporally extended energy bands associated with module-level EM coupling, HFCT signals appear as sporadic, short-duration high-frequency bursts, albeit partially attenuated and temporally dispersed due to current sharing among series-connected cells.

Compared to single-cell measurements, the module-level responses exhibit reduced impulsive sharpness and enhanced low-frequency content, attributable to electrical averaging, increased parasitic inductance, and cell-to-cell imbalance effects inherent to series configurations. These results demonstrate that, under moderate operating conditions, module-level diagnostics shift the dominant signal characteristics from highly localized cell-internal events to system-level EM behavior, underscoring the necessity of combined EM antenna and HFCT sensing to bridge the diagnostic gap between cell- and module-scale battery health monitoring.

### 4.3. Comparative Analysis and Discussion

In this study, “wavelet energy” denotes a signal-processing metric defined as the squared magnitude of wavelet coefficients. It does not represent physical electrical energy in joules, since the measurement is performed on a complex RLC-equivalent electrochemical system without phase-resolved voltage–current information. Therefore, it is employed solely as a relative amplitude indicator for transient intensity comparison across scales, independent of impedance assumptions.

The FFT spectrum is expressed in dB re 1 mV, representing the logarithmic voltage magnitude normalized to a 1 mV reference, which enables quantitative comparison of high-frequency transient amplitudes associated with PD and MISC phenomena.

The apparent discrepancy between the dominant frequency bands observed in FFT spectrum and Morlet CWT arises from the fundamental difference between Fourier-based and wavelet-based signal analysis. The FFT spectrum represents a time-averaged frequency distribution over the entire measurement window, including both PD events and non-discharge intervals. As a result, the transient high-frequency components generated by PD are spread over a broad frequency range and appear attenuated toward lower frequencies.

In contrast, the Morlet CWT time–frequency spectrum provides a time–frequency localized representation, allowing the instantaneous energy of PD events to be isolated. The high-energy regions observed in the 3–5 MHz band correspond to the actual emission frequencies of PD occurring within a short temporal duration. Therefore, the frequency bands highlighted by the color intensity in Morlet CWT spectrum represent the true localized EM radiation characteristics of PD events, rather than a time-averaged spectrum.

Consequently, the difference in frequency distribution between FFT spectrum and Morlet CWT spectrum does not indicate inconsistency, but rather demonstrates the complementary nature of FFT and CWT analyses for non-stationary PD signals in LIBs.

To provide a concise comparative overview of the dominant signal characteristics observed under different operating conditions and sensing modalities, the key trends extracted from the DWT and CWT analyses are summarized in Table 6.

As summarized in Table 6, EM antenna measurements predominantly capture low-to-mid-frequency electromagnetic emissions associated with system-level dynamics and global electrochemical imbalance, whereas HFCT measurements emphasize high-frequency, sharply localized transient signatures linked to internal current fluctuations. Under high C-rate and elevated temperature conditions, the dominance of high-frequency DWT levels (D1–D2) becomes increasingly pronounced in HFCT measurements, supporting the hypothesis that degradation-induced MISC manifests as impulsive, broadband electrical events. Considering the 20 MHz sampling rate, the first DWT decomposition level (D1) approximately corresponds to the 5–10 MHz band, whereas higher levels represent progressively lower frequency ranges. At the module level, a shift toward lower-frequency dominance and reduced impulsive sharpness is observed due to current redistribution and parasitic impedance effects inherent to series-connected configurations.

Under high-stress conditions (5C, 60 °C), the D1 energy ratio increased to approximately 0.38–0.52 of total wavelet energy, compared to less than 0.12 under moderate operating conditions. The transient amplitude threshold for event detection was set to Max 180 mV to Min −150 mV, above which impulsive signatures were consistently associated with degradation-induced MISC-like behavior.

In terms of qualitative repeatability, consistent frequency-band concentration trends were observed across repeated experiments and varying stress conditions. Although the absolute transient amplitudes and event densities exhibited moderate variability depending on temperature, C-rate, and aging progression, the dominant frequency localization and relative wavelet energy concentration patterns remained stable. In particular, high-stress conditions consistently showed enhanced D1-level energy concentration in HFCT measurements and low-to-mid-frequency dominance in EM antenna measurements. These trends were reproducible across repeated cycling tests and multiple cells/modules, indicating that the identified time–frequency signatures represent systematic degradation-related phenomena rather than random measurement artifacts. While cycle-to-cycle variations were present due to stochastic nature of MISC events, the overall frequency-band concentration characteristics remained qualitatively robust.

Future work may incorporate simultaneous voltage–current acquisition to enable impedance-aware transient power estimation and phase-resolved analysis for enhanced physical interpretability.

### 4.4. Real-Time Implementation Considerations

The practical feasibility of implementing the proposed diagnostic framework in real-time BMS was also considered. Among the two wavelet techniques employed in this study, the DWT is particularly suitable for embedded implementation due to its low computational complexity (O(N)) and filter-bank-based structure, which can be efficiently realized on field-programmable gate array (FPGA) or digital signal processor (DSP) platforms. In contrast, the CWT, while valuable for detailed offline time–frequency interpretation, requires significantly higher computational resources and is therefore more appropriate for laboratory analysis and model validation rather than real-time deployment.

From a hardware perspective, the sampling rate represents a critical design trade-off between transient resolution and system cost. Although microsecond-level sampling (e.g., 0.050 μs) enables accurate capture of high-frequency impulsive signatures up to 10 MHz, practical BMS implementations may adopt optimized bandwidth selection or adaptive down-sampling strategies to reduce data throughput while preserving diagnostically relevant features. The associated memory footprint can be minimized by processing signals in short sliding windows and storing only wavelet energy coefficients instead of raw waveform data.

For real-time fault indication, a threshold-based energy detection logic can be implemented by monitoring the relative wavelet energy ratio in selected decomposition levels (e.g., D1/D_total or D2/D_total). When the energy concentration exceeds a predefined adaptive threshold, the system can trigger an early warning flag for potential MISC-related anomalies. Such an approach allows computationally efficient event detection without requiring full-scale spectral reconstruction.

Overall, the proposed DWT-based multi-sensor framework can be integrated into a BMS microcontroller unit (MCU) with moderate processing capability, enabling scalable, real-time monitoring of degradation-induced transient signatures for proactive battery safety management.

## 5. Conclusions

In this study, an integrated sensor-based diagnostic framework combining low-frequency EM antenna sensing and HFCT sensing was systematically investigated for early fire risk prediction in LIBs at both cell and module levels.

This study experimentally demonstrated that degradation-induced MISCs generate distinct non-stationary electrical signatures that can be effectively captured by complementary EM antenna and HFCT sensors.

Extensive experiments conducted under diverse operating conditions—including varying C-rates, ambient and elevated temperatures, and different stages of degradation—demonstrated that degradation-induced electrical anomalies manifest as distinct, non-stationary signatures across multiple frequency domains.

Unlike conventional safety indicators, the proposed wavelet-based approach enables early-stage detection of incipient internal faults prior to observable thermal or capacity degradation.

At the cell level, high-stress conditions such as high C-rate and elevated temperature produced pronounced impulsive high-frequency transients captured by the HFCT sensor, which were strongly localized in time and dominated the lowest wavelet decomposition levels, indicating discharge-like events associated with incipient internal defects. In contrast, the EM antenna sensor predominantly captured low- to mid-frequency EM emissions reflecting global electrochemical imbalance, current redistribution, and system-level EM coupling.

At the module level, these signatures exhibited reduced impulsive sharpness and increased low-frequency dominance due to current sharing, parasitic impedance, and cell-to-cell imbalance inherent to series-connected configurations, highlighting a clear scale-dependent transformation of degradation signals from localized to spatially averaged behavior.

By modeling the LIB as an electrical insulation system with the separator acting as the primary dielectric, the observed electrical signatures were consistently interpreted as PD-like transient electrical signatures associated with MISC phenomena in LIBs, rather than conventional gas-insulated partial discharge events. The combined application of CWT and DWT enabled robust time–frequency localization and multi-scale energy characterization, allowing effective phenomenological differentiation between normal operational noise and defect-related precursors. Therefore, this work primarily serves as an experimental and phenomenological investigation into the time–frequency characteristics of degradation-induced PD-like signatures associated with MISC, establishing a physically grounded foundation for subsequent quantitative validation and real-time diagnostic algorithm development.

Overall, the results confirm that the proposed multi-sensor, wavelet-based approach provides complementary and scalable physically interpretable diagnostic potential across cell and module levels, offering a physically grounded and practically deployable solution for early detection of degradation and fire-related risks in advanced LIB systems.

The proposed framework can be directly integrated into advanced battery management systems for real-time safety monitoring in electric vehicles and large-scale energy storage systems.

Future work will focus on extending the proposed diagnostic framework to a broader range of battery chemistries and form factors, including pouch-type, prismatic, and solid-state batteries, in order to validate the universality and robustness of the identified frequency-domain indicators. In addition, degradation experiments under low-temperature operating conditions (<0 °C) will be conducted to investigate temperature-dependent variations in electrical signature characteristics and to assess the applicability of the method under cold-climate scenarios. Furthermore, the integration of artificial intelligence (AI)-based classification and pattern recognition techniques will be explored to enable automated detection of MISC events and to further enhance diagnostic accuracy, reliability, and scalability for real-time battery health monitoring systems.

## Figures and Tables

**Table 1:** Differences in pattern analysis between CWT and DWT.

Category	CWT	DWT
Scale	Continuous	Discrete
Time Shift	Continuous movement	Discrete shift
Output	Time-scale map	Coefficient vector
Resolution	Very detailed	Stage-wise fixed
Computational Amount	High	Low
Computational Speed	Slow	Fast
Pattern Shape	Image-based (map, contour)	Numerical (coefficient, energy)
Real-Time Analysis	Difficult	Possible

**Table 2:** DWT and CWT MW selection strategy (based on study design).

Research Purpose	Recommended Transform	Recommended MWs
Real-time diagnosis	DWT	db4, sym4
Feature vector extraction	DWT	db6, coif5
Phenomenological interpretation	CWT	Morlet
Early detection of PD/MISC	CWT	Morlet
High frequency transient	CWT	Morlet/Paul
Signal compression	DWT	bior

**Table 3:** Key Characteristics of MISC and hard ISC.

Feature	MISC	Hard ISC
Short-circuit resistance	High (mΩ–Ω)	Very low
Current magnitude	mA–A	Hundreds of A
Temporal behavior	Intermittent	Continuous
Detectability	Difficult	Easy
Role in TR	Precursor	Direct trigger

**Table 4:** Critical Temperature Thresholds.

Reaction	Temperature Range
SEI breakdown	90–120 °C
Separator melting	130–160 °C
Electrolyte oxidation	150–200 °C
Cathode oxygen release	>200 °C

**Table 5:** Cylindrical battery specifications.

Name	Samsung INR18650-30Q (Samsung SDI; Yongin-si, Gyeonggi-do, Republic of Korea)
Norminal capacity	3000 mAh
Norminal voltage	3.6 V
Charge cut-off voltage	4.2 V
Discharge cut-off voltage	2.5 V

**Table 6:** Comparative Summary of Dominant Signal Characteristics.

Condition	Sensor	Dominant Frequency Band	Dominant DWT Level	Signature Characteristics
CCCV initiation (system transient)	EM Antenna	5–6 MHz (transition between D1–D2 bands)	D1–D2	Dual control-loop transient burst
1C, 60 °C (Cell)	EM Antenna	~1 MHz	D4–D8	Repetitive PD-like voltage spikes
1C, 60 °C (Cell)	HFCT	9–10 MHz	D1	Sharp impulsive current transients
2C, 25 °C (Cell)	EM Antenna	<4 MHz	D6–D8	Sustained low-frequency EM emissions
5C, 60 °C (Cell)	EM Antenna	Broad (1–4 MHz)	D4–D7	High-amplitude broadband EM fluctuation
5C, 60 °C (Cell)	HFCT	8–10 MHz	D1–D2	Localized discharge-like bipolar spikes
2C, 25 °C (Module 4S1P)	EM Antenna	<3 MHz	D5–D7	Spatially averaged low-frequency behavior
2C, 25 °C (Module 4S1P)	HFCT	7–9 MHz	D1–D3	Attenuated high-frequency transient bursts

## Data Availability

The original contributions presented in this study are included in the article. Further inquiries can be directed to the corresponding author.

## Abbreviations

AI	Artificial intelligence
CC	Constant current
CCCV	Constant current–constant voltage
CMV	Common mode voltage
CWT	Continuous wavelet transform
DSP	Digital signal processor
DWT	Discrete wavelet transform
EM	Electromagnetic
ESSs	Energy storage systems
EVs	Electric vehicles
FFT	Fast fourier transform
FPGA	Field-programmable gate array
HFCT	High-frequency current transformer
ISC	Internal short circuit
LIBs	Lithium-ion batteries
LVDC	Low-voltage direct current
MCU	Micro-controller unit
MISC	Micro internal short circuits
MRA	Multi-resolution analysis
MW	Mother wavelet
PD	Partial discharge
SEI	Solid electrolyte interphase
TR	Thermal runaway
WT	Wavelet transform
